# Promotion focus, but not prevention focus of teachers and students matters when shifting towards technology-based instruction in schools

**DOI:** 10.1038/s41598-024-73463-z

**Published:** 2024-09-25

**Authors:** Emely Hoch, Katharina Scheiter, Kai Sassenberg

**Affiliations:** 1 Leibniz-Institut für Wissensmedien, Schleichstraße 6, 72076 Tübingen, Germany; 2 University of Potsdam, Potsdam, Germany; 3 Leibniz-Institute for Psychology (ZPID), Trier, Germany

**Keywords:** Psychology, Human behaviour

## Abstract

Implementing technology-enhanced learning and teaching in schools causes major changes for both teachers and students. Research in other fields shows that individuals’ regulatory orientation (i.e., promotion and prevention focus) predicts how people deal with such fundamental changes. Our research ties in with a school trial where classes were equipped with tablets, yielding a shift from predominantly analog to technology-enhanced learning and teaching. In this context, we investigated whether the regulatory orientation of teachers (*N* = 109) and students (*N* = 1,127) related to how they dealt with this change. As expected, teachers with a stronger promotion focus were more likely to perceive the changing teaching situation as a (positively connotated) challenge and to intend to use the tablets in class. Further, students with a strong promotion focus when tablet-based teaching was introduced showed higher technology-related self-efficacy and higher motivation to use digital devices after a few months of using tablets in class. Prevention focus was unrelated to teachers’ and students’ attitudes, behavioral intentions, or motivation. Thus, the concept of regulatory orientation helps explain how teachers and students respond to changes in school, whereby particularly promotion focus appears to be relevant for change processes due to digital transformation in education.

## Introduction

Using technologies in the classroom holds various promises for teaching and learning^1,2^. However, reality can only live up to these promises when teachers and students adapt to the required changes in classroom practices^3–5^. We put forward that how one copes with such changes also depends on their regulatory orientation^6,7^, that is, which motivational system is primarily activated: promotion or prevention focus. Research with university students and employees has shown that people focusing on ideals, advancement, and gains—a so-called promotion focus^6^—are more likely to deal with change and new situations successfully^8–10^.

To date, little is known about the determinants of successful change in schools, such as the adoption of technology^1^. When introducing technology in schools, regulatory orientation may explain how well teachers and students deal with this change in their classroom practices. We tested this assumption in the context of a governmental initiative in Germany, where teachers and students in secondary education were equipped with tablet computers on a one-to-one basis. In particular, we investigated whether teachers’ regulatory orientation prior to the introduction of tablet computers related to their intention to adopt technology-enhanced teaching practices (study 1). Further, we investigated whether students’ regulatory orientation prior to receiving the tablet computers related to how they perceived the tablet computers after having worked with them for some months (study 2).

## Educational technology in the classroom (in Germany)

Research has shown that—if used adequately—educational technologies effectively promote learning and understanding^1^. Accordingly, using technology in the classroom is considered an important innovation in nowadays teaching and learning^11^. However, despite the omnipresence of technology in our daily lives, students and teachers are yet rather unfamiliar with technology-supported teaching, particularly in Germany^1,12^. Notably, Germany has fallen behind regarding the use of digital devices in the classroom, as reflected in a lack of available hardware, poor infrastructure, and limited use of technology for teaching^11,12^. According to the *International Computer and Information Literacy Study 2018* (ICILS)^12^, in Germany, on average, ten students share one digital device (e.g., compared with two students sharing one digital device in the US) and, for instance, learning management systems or digital learning resources for online use are lacking. Furthermore, in Germany, there is a lag regarding access to wireless LAN in international comparison, and increasing bandwidth of internet access is reported as one of the priorities to improve the situation in German schools. Consequently, the Ministers of Education and Cultural Affairs decided on a national strategy to increase the use of technology in classrooms^13^, which eventually led to federal and state-wide initiatives aimed at equipping schools with digital devices (e.g., in one-to-one programs) and the necessary infrastructure.

## How the use of digital devices changes classroom practices

According to a meta-analysis, one-to-one laptop programs change classroom practices, reflected in an increased use of technology for learning purposes, and improve academic achievement^14^. When switching to technology-enhanced teaching, instructors are required to redesign and restructure teaching and learning activities to benefit from the technologies’ educational potentials^15^. This might be accompanied by some uncertainty in the classroom (for teachers and students), as teachers cannot draw on their expertise in traditional teaching but must develop new teaching routines. Similarly, students may be unfamiliar with these new practices while also adjusting the way they learn (e.g., project-based learning)^14^. Teaching with digital devices is often associated with more freedom and self-regulated learning scenarios for students. Thus, compared with a more traditional classroom setting with teacher-centered instruction, technology-enhanced teaching often induces a shift towards more individualized and student-centered pedagogies^1,14,16–18^. Instead of being the direct instructor and functioning as key masters to dispense knowledge, teachers are there to design learning contexts; they act as consultants, moderators, and supporters, thereby becoming facilitators for knowledge acquisition. This implies that students are encouraged to take more responsibility for their learning processes. For instance, they are expected to explain or discuss their ideas with one another online or collaborate in remote problem-solving or constructive tasks^2,19^. Thus, introducing digital devices to the classroom marks an educational change, which means that teachers and students need to get used to new teaching and learning methods and explore how they can use digital devices effectively.

## Regulatory focus theory

*Regulatory Focus Theory* is one theory that makes predictions about how people approach novel and potentially challenging situations^6,7,20^. The theory is concerned with selecting means (i.e., strategies) that individuals apply consciously or unconsciously when striving towards desired end states. It connects motivation and how people approach a goal or demand at hand. The regulatory focus theory postulates two distinct regulatory orientations: promotion focus and prevention focus. Individuals with a promotion focus are concerned about advancement, growth, and accomplishment. They use eager goal-striving strategies, like trying out new things, and are sensitive to the presence and absence of positive outcomes (gain vs. non-gain). When facing an exam, students with a strong promotion focus would, for instance, anticipate the pleasure of receiving an A. In contrast, individuals with a prevention focus are concerned with protection, safety, and responsibility. They apply a defense strategy in goal striving; that is, they carefully avoid making errors, follow rules, and are sensitive to the presence and absence of negative outcomes (loss vs. non-loss). When facing an exam, students with a strong prevention focus would, for instance, anticipate the pain of receiving less than an A.

The distinction between the two foci most relevant to the current context is that people with a promotion focus like to try out new things and seek out challenges, whereas people with a prevention focus instead prefer well-known grounds where they can follow given rules and more easily avoid errors^6^. In line with this notion, a series of studies demonstrated that a stronger promotion focus is associated with higher openness for and willingness to change, whereas a stronger prevention focus is associated with a stronger preference for stability and less willingness to change^8,10^.

There is extensive evidence for the predictions of regulatory focus theory in experimental, work, organizational, and health psychology (for an overview, see^21^). However, it has rarely been applied within educational contexts (for exceptions, see, e.g.,^22–24^). Given that achievement and accomplishment, as well as rules and avoiding errors play a crucial role in educational settings, we assume that regulatory focus theory contributes to understanding how students and teachers cope with changes in school as an educational setting and workplace.

## The present research

In two studies, we tested the idea that regulatory focus theory can be applied in an educational context and contribute to understanding how teachers and students cope with switching to technology-enhanced teaching and learning. Thus, we investigated the role of regulatory orientation in dealing with a substantial change in classroom practices arising from the introduction of tablet computers. Based on prior research in related fields, we hypothesized that a stronger promotion focus would relate to more positive attitudes, and a stronger prevention focus would be associated with less positive attitudes towards the introduction of technology in the classroom. For this purpose, teachers (study 1) and students (study 2) of school classes were examined that were part of a school trial in which students were equipped with tablet computers on a one-to-one basis.

## Study 1: Teachers

Teachers have been shown to deal with and respond to educational change differently depending on their individual dispositions^25^, thereby being key to determining the course of a change process^26^. Previous research on how teachers’ perceptions of technology affect their intentions and actual use of technology for teaching (e.g.,^5,27,28^) is most often conducted against the backdrop of the Technology Acceptance Model (TAM) and its derivates^29^. It shows that teachers’ intention to use technology in the classroom can be explained by the way they perceive technology, that is, its usefulness and ease of use (for meta-analyses, see^30,31^). However, these variables tend to be very specific to each technology used. We see merit in taking a step away from such proximal variables and looking at more distal predictors. These might allow for a prediction of teachers’ readiness to adapt across a diverse range of technologies, which is more in line with the actual situation in schools. In this light, we examined teachers’ general approach toward change by examining their regulatory orientation.

Against the backdrop of regulatory focus theory^6^ and meta-analytic evidence showing that regulatory orientation is related to work behaviors, job engagement, and attitudes^9^, we aimed to investigate whether teachers’ regulatory orientation is linked to their willingness to change their classroom practices in response to introducing technology (RQ1). To this end, we tested whether teachers’ regulatory orientation, assessed before their classes were equipped with tablet computers on a one-to-one basis, relates to their intention to actually use the to-be-introduced tablet computers in everyday teaching. Because individuals with a promotion focus are more open to change and like to try new things, we expected a positive relation between teachers’ promotion focus and their intention to use tablet computers in the classroom (hypothesis 1a). Because individuals with a prevention focus prefer stability and are less willing to implement change, we expected a negative relation between teachers’ prevention focus and the intention to use tablet computers in the classroom (hypothesis 1b).

Further, we explored the relation of regulatory orientation with how teachers perceive the expected change. The demand to change classroom practices might be perceived either as an opportunity that appears challenging in a positive sense or as a responsibility that appears threatening. Introducing technology to the classroom opens up the opportunity for teachers to try new teaching methods (e.g., simulate real-world situations in the classroom)^32^ that would not have been possible without technology. At the same time, the downside of switching to new classroom practices is that it might initially overwhelm teachers by requiring technical and pedagogical skills not yet acquired^33^. Accordingly, some studies reported that teachers have initial concerns about technology-enhanced instruction (cf.^14^); moreover, training teachers to use digital tools led to stronger effects of technology on student achievement in a recent meta-analysis^1^. Thus, changing classroom practices can be construed as opportunity and challenge (focusing on potentials and opportunities that new classroom practices offer) or as responsibility and threat (focusing on pitfalls and uncertain situations resulting from lack of experience with the new classroom practices).

Construal of change as opportunity or responsibility is closely related to individuals’ regulatory orientation (for similar reasoning, see^34,35^) and to the perception of demands as threat and challenge^36^. Individuals with a strong promotion focus are more likely to construe change as an opportunity with which they are challenged as they are more open to change, eager to try out new things, and have higher aspirations; individuals with a strong prevention focus, on the other hand, more likely construe change as a responsibility to which being obliged as they prefer stability and avoid making errors^10^. For them, the demands due to the change are more likely to appear threatening^37^.

Against this backdrop, we investigated how teachers’ regulatory orientation relates to perceiving the demands of changing classroom practices as either challenging or threatening (i.e., less or more stressful; RQ2). We assumed that the stronger teachers’ promotion focus is, the more likely they are to perceive changing classroom practices as a challenge (hypothesis 2a). Further, the stronger the teachers’ prevention focus is, the more likely they are to perceive changing classroom practices as a threat (hypothesis 2b).

### Method

#### Context of the study

The research project *tabletBW meets science* ties in with the school trial *tabletBW*, which was initiated by the federal state of Baden-Württemberg, Germany. The school trial aimed to promote technology-based teaching and learning. Secondary schools (only Gymnasium, the highest track of German high school) from Baden-Württemberg could apply to become part of the school trial and receive financial support to equip four classes with tablet computers. From all applicants, 14 schools were randomly chosen. Students and their teachers from two classes of 7^th^ grade in two cohorts (i.e., two classes of 7^th^ grade in two consecutive years, thus, four classes per school) were equipped with tablet computers on a one-to-one basis, resulting in 56 classes that participated in the school trial. Teachers in the tablet classes were asked to integrate tablet computers into their teaching practices; however, they were not required to do so, nor were there any mandatory training programs or introductions on how to use tablet computers in their teaching. An equal number of classes formed the control group for the research project. These classes were of another 14 randomly chosen schools that had also applied for the school trial but had not been considered for being equipped with tablet computers as part of the school trial. The classes from the 14 tablet schools and the 14 control schools formed the sample for the research project. Because the data was collected in the context of an initiative aimed at equipping schools with tablets, teachers generally had little prior experience with teaching with tablets (for more details, see online supplementary materials of^38^). Institutional approval for the study design, the study contents, study materials and the data protection procedure was received from the Ministry of Cultus, Youth, and Sports Baden-Württemberg. The procedures followed the APA guidelines (e.g., voluntary participation, informed consent)^39^. Teachers and students’ participation was voluntary. Given that informed consent was provided, data were assessed from teachers, students, and their parents for three years. When the school trial and research project were conducted, tablet computers or other digital devices were scarcely available in German classrooms. Some schools had devices that teachers could borrow to bring to the classroom for individual lessons. Hence, one-to-one equipment represented an opportunity to redesign everyday teaching fundamentally.

This study was conducted as part of the research project *tabletBW meets science*. As the current study addressed how individuals cope with the change in classroom practices due to introducing technology, we only considered data from participating tablet classes, while data from control classes were not considered. Study 1 uses data from teachers who participated the first time at baseline measurement, that is, before they started working with the tablet computers in class (i.e., spring 2018 in cohort 1 and spring 2019 in cohort 2).

#### Participants

**Fig. 1:**
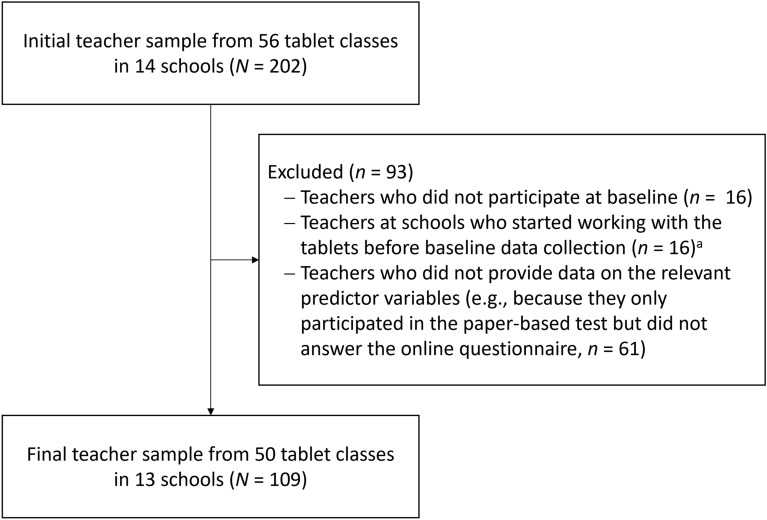
Teacher participant flowchart. (^a^Teachers of these classes were excluded because the change we were interested in had already taken place in these schools; the exclusion did not change the pattern of results.)

A total of 202 teachers from tablet classes participated in the data collections of the overall project. After applying our exclusion criteria (for teacher participant flow, see Fig. 1), the sample we analyzed comprised data from *N* = 109 teachers (57 female, 52 male) who were 24 to 62 years old (*M* = 37.78 years, *SD* = 7.89) and had been working as teachers for 1 to 34 years (*M* = 10.12 years, *SD* = 7.31; two teachers did not provide duration in teaching profession).

#### Measures

All items of the teachers’ questionnaire scales relevant to this study are listed in Appendix A.

Regulatory orientation was assessed as promotion focus (12 items, Cronbach’s α = 0.75, e.g., “I am guided by my ideals.”) and prevention focus (eight items of which two items were reversely coded, Cronbach’s α = 0.63, e.g., “In case of important decisions, security is a core criterion I care for.”) on a 5-point scale ranging from (1) *not at all* to (5) *very much* (adapted from^34^).

Teachers’ intentions to use tablet computers in their teaching were measured with five self-developed items. For the question “How would you rate your intentions to use tablet computers in the classroom?” teachers were asked to rate five statements (Cronbach’s α = 0.68, e.g., “I will look for meaningful ways to use tablet computers in my classroom in the future.”) on a 4-point scale ranging from (1) *not at all* to (4) *very much*.

We adapted the stress appraisal measure to assess whether teachers construe the switch to teaching with tablet computers as a challenge or threat^40^. Teachers were first presented with the following instruction: “Starting in the coming school semester, you will be expected to use tablet computers in your lessons. How much do you agree with the following statements regarding this situation?” which was followed by 14 statements to be rated on a 4-point scale ranging from (1) *not at all* to (4) *very much*. With seven items each, the extent to which teachers perceived this situation as a challenge (Cronbach’s α = 0.69, e.g., “I can use the high demands positively for my purposes.”) or a threat (Cronbach’s α = 0.88, e.g., “I am afraid of not being able to cope with this situation.”) was measured (four teachers did not provide data for the stress appraisal measure scale).

#### Procedure

Given that informed consent was provided, teachers were provided with the materials of the research project. They could decide for themselves when to work on the questionnaires and tests as long as they submitted their responses by a specified date. The comprehensive self-report questionnaires were completed online and comprised questions on teachers’ background (e.g., demographics, teaching experience), attitudes and beliefs about teaching in general (e.g., self-efficacy in teaching, teaching quality), and experience with and attitudes towards computers and tablet computers (e.g., computer and tablet computer use professionally and privately). Further, online and paper–pencil tests assessed teachers’ skills and knowledge (e.g., pedagogical knowledge). The scales relevant to the current research (i.e., regulatory orientation, intentions to use tablet computers, stress appraisal measure) were all assessed in the online self-report questionnaire. Completing the questionnaire and tests, in total, took about 90 min.

### Results

All analyses were conducted with the statistical software R version 4.3.1^41^ and the lavaan package version 0.6–15^42^. Descriptive values (means and standard deviations) and correlations of all study variables are reported in Table 1.

**Table 1:** Descriptive statistics (sample size, mean, standard deviation) and zero-order correlations for all study variables of the teacher sample.

	*n*	*M*	*SD*	1	2	3	4
1. Promotion focus	109	3.66	0.43	−			
2. Prevention focus	109	3.68	0.49	− 0.16	–		
3. Intentions to use tablet computers	109	3.36	0.44	0.38***	− 0.10	–	
4. Challenge	105	2.99	0.39	0.32***	−0.22*	0.44***	–
5. Threat	105	1.59	0.55	− 0.29**	0.13	− 0.32**	− 0.31**

*Note*. Promotion and prevention focus were assessed on a 5-point scale; challenge, threat, and intentions to use tablet computers were assessed on a 4-point scale.

***p <0 .001, **p < 0.01, *p < 0.05.

We fitted a multivariate regression model (see Table 2) to answer research questions 1 and 2. Promotion and prevention focus were included as predictor variables; intentions to use tablet computers in classroom teaching, construal of change as challenge, and construal of change as threat served as criterion variables while controlling for the teachers’ age and gender (female = 0, male = 1). No interaction terms were included in the model. The data to fit the model was cross-sectional, with all variables having been assessed at baseline, that is, prior to starting to work with the tablet computers in classroom lessons. The parameters were calculated using maximum likelihood estimation using the Yuan-Bentler correction and cluster robust standard errors with school as cluster variable (for a strong recommendation to use this approach, see^43^). Further, we estimated the variances and covariances of the exogenous variables in the model to account for these variables when estimating missing values in the full information maximum likelihood procedures for missing data.

**Table 2:** Fitted model of the teacher sample.

Predictor	Dependent variables								
	Intentions to use tablet computers			Challenge			Threat		
	*Est*	*SE*	*p*	*Est*	*SE*	*p*	*Est*	*SE*	*p*
Promotion focus	0.381	0.088	** < 0.001**	0.230	0.082	**0.005**	− 0.310	0.231	0.181
Prevention focus	− 0.027	0.153	0.858	− 0.099	0.079	0.210	0.020	0.146	0.890
Gender	0.030	0.062	0.630	0.142	0.065	**0.029**	− 0.235	0.082	**0.004**
Age	0.001	0.006	0.905	− 0.005	0.006	0.365	0.003	0.006	0.593
*R*^*2*^	0.145			0.160			0.125		

*Note. N* = 109. Significant *p*-values (*p* < 0.05) are shown in bold. Perfect model fit due to saturated model. The pattern of results for individual regression models for the three dependent measures was the same, and the parameters were almost identical.

#### RQ1: Is teachers’ regulatory orientation related to their intentions to use tablet computers in the classroom?

We hypothesized that teachers’ promotion focus would be positively associated with their intentions to use tablet computers in the classroom (hypothesis 1a) and that teachers’ prevention focus would be negatively associated with their intentions to use tablet computers in the classroom (hypothesis 1b). Results demonstrated that promotion focus related positively to teachers’ intention, whereas prevention focus did not show a significant association (Table 2). Thus, these results supported the hypothesis regarding a positive relation of promotion focus with the intention to use tablet computers in the classroom (hypothesis 1a) but were not in line with the hypothesis regarding a negative relation of prevention focus (hypothesis 1b).

####  RQ2: Is teachers’ regulatory orientation related to their perception of change as a challenge or threat?

We hypothesized that the stronger teachers’ promotion focus, the more likely they would construe change as a challenge (hypothesis 2a), and the stronger teachers’ prevention focus, the more likely they would construe change as a threat (hypothesis 2b). Results demonstrated that in line with hypothesis 2a, promotion focus positively related to the construal of change as a challenge (see Table 2). In contrast, prevention focus was not significantly associated with the construal of change as a threat, thereby not aligning with hypothesis 2b.

Additionally, significant gender differences emerged for the construal of change as a challenge or a threat. Compared to female teachers, male teachers were more likely to construe the change to technology-enhanced teaching as a challenge and less likely to construe it as a threat.

### Discussion

Study 1 shed light on how teachers dealt with changing teaching practices from switching to technology-enhanced teaching. In line with our hypothesis, the stronger the promotion focus was, the more willing teachers were to change their teaching and use tablet computers in their classroom teaching. Further, we found evidence that teachers with a stronger promotion focus more likely construed the change to tablet-based teaching as a challenge.

Unexpectedly, we found no evidence for an association between prevention focus and teachers’ intention to use tablet computers, nor for them construing this change as a threat. While this hypothesis was derived from theory and in line with prior research findings^6,21,44^, previous research also showed that the results for prevention focus are less consistent compared with results for promotion focus^9^. For instance, in two meta-analyses that also looked at work-related outcomes, promotion focus was shown to be positively associated with task performance, whereas counter to theory-based expectations, prevention focus was unrelated to task performance^9,44^.

Another explanation for the missing relation of prevention focus to the intention to use tablet computers in classroom teaching could pertain to social desirability. The schools participating in the research project were drawn from applicants for a school trial initiated by the Ministry. The fact that the schools had applied for the equipment suggests that there was at least a desire, if not a demand, for technology-enhanced instruction from the school’s organizational side. This is worth mentioning as a stronger prevention focus goes hand in hand with the urge to fulfill obligations^6^. Therefore, a strong prevention focus could be accompanied by a lower intention to use tablet computers because of the desire for stability on the one hand and a higher intention to use tablet computers because of the urge to fulfill obligations set by the school board on the other hand. Nonetheless, one would still expect that teachers with a strong prevention focus would construe such a change as a threat, which we did not show either.

It is important to note that the introduction of technology not only changes for teachers but also affects students who are not yet familiar with learning with technology. How students dealt with these changes depending on their regulatory orientation was addressed in a longitudinal design in study 2.

## Study 2: Students

Students are in a different situation than teachers since they typically are not to decide whether to use technology when it is introduced into their daily classroom teaching practices. The literature mainly reports positive attitudes of students towards technology in the classroom^14,45^. Nevertheless, students’ attitudes toward technology and openness toward accepting this change in teaching may still affect their learning behavior, engagement, and motivation to learn.

In a broad sense, motivation is defined as being moved to do something which, consequently, gives rise to an action^46^. It can be specified as a preference toward specific activities, objects, or events and refers to liking, value, and interest in a task or activity. Research showed that, in general, interest is associated with improved learning^47,48^; a recent meta-analysis confirmed interest as a predictor of academic achievement^49^. Regarding technology, in particular, it has been shown that students’ attitudes toward technology and how they perceive educational technology drive their intention and actual use of it^50,51^.

How well students think they can use technology may as well be related to their learning behaviors in technology-enhanced teaching. Self-efficacy beliefs refer to beliefs in one’s abilities to perform a given behavior^52^, that is, individuals’ conviction that they can successfully execute behaviors necessary to produce specific performance outcomes. Evidence shows that self-efficacy is associated with academic achievement and performance^53–56^. Students’ self-efficacy in using technology has been shown to relate to their computer and information literacy^57,58^ and to better grades in online courses^59^. Thus, understanding motivation in educational settings is highly relevant because of the consequences for learning behavior and its relation to student achievement^54,60,61^.

The regulatory focus theory describes motivational consequences that may arise from how individuals cope with novel situations. Given that promotion and prevention focus imply preferences for certain types of situations or tasks—i.e., individuals with a stronger promotion focus like to try out new things, and individuals with a stronger prevention focus prefer to follow given rules^6^—research has studied the influence of the fit between the implied preferences of regulatory orientation on the one hand and the specific task on the other hand. According to the regulatory fit hypothesis^20,62^, engagement and motivation are stronger when a situation, event, or behavior fits an individual’s regulatory orientation. In addition, the situation, objects, and people an individual interacts with will be perceived more positively in case of a high regulatory fit^63,64^. Further, high regulatory fit goes along with higher engagement and effort for fitting tasks^65^. For example, a person with a promotion focus will feel more positively and engage more strongly when writing an essay on a topic of one’s own choice than when writing dictation because the essay task allows one to make more use of the preferred strategy. On the other hand, a person with a prevention focus will feel more positively and engage more strongly with the dictation because it fits the preference to follow given rules.

As described above, technology-supported teaching is characterized by more individualized and student-centered pedagogies where students take a more active and self-regulated role in their learning^1,2,14,16,18^. Such an environment is characterized by more degrees of freedom for students and less tight teacher-directed instructional practices. Therefore, it can be assumed that students’ strength of engagement and motivation differs depending on the fit of technology-enhanced teaching and learning situation to students’ regulatory orientation.

In the current research, we aim to investigate whether students’ regulatory orientation relates to their motivational outcomes in the context of introducing technology in the classroom. For this purpose, we used longitudinal data from students from the school trial in which 7^th^-grade students were equipped with tablet computers on a one-to-one basis. Specifically, we examined whether students’ regulatory orientation measured prior to handing out the tablet computers at the beginning of the school term is associated with motivational variables (i.e., their perception of technology, technology-related self-efficacy, and motivation to use technology) assessed at the end of the school term. In particular, we expected students with a strong promotion focus to respond more positively to the experience of learning with tablet computers in the classroom than those with a high prevention focus for at least two reasons. First, they are expected to be more open to change; second, the situation better fits their regulatory orientation. Thus, after having worked with the tablet computers in class for some months, we expected students with a stronger promotion focus to perceive technology more positively (hypothesis 1a), to have higher technology-related self-efficacy (hypothesis 2a), and to be more motivated to use technology (hypothesis 3a). In turn, we expected no or even negative effects on motivational outcomes for students with a stronger prevention focus due to their preference for well-known grounds and low fit to the situation. Thus, after having worked with the tablet computers in class for some months, we expected students with a stronger prevention focus to perceive technology less positively (hypothesis 1b), to have lower technology-related self-efficacy (hypothesis 2b), and to be less motivated to use technology (hypothesis 3b).

### Method

#### Context of the study

Data stem from the same school trial as Study 1. Study 2 refers to data from students at the time when being equipped with tablet computers (t_0_, i.e., at the end of the first school semester of 7^th^ grade, which was in spring 2018 and 2019 in cohort 1 and 2, respectively) and approximately four to five months later (t_1_, i.e., at the end of the second school semester of 7^th^ grade, which was in summer 2018 and 2019 in cohort 1 and 2, respectively).

#### Participants

**Fig. 2:**
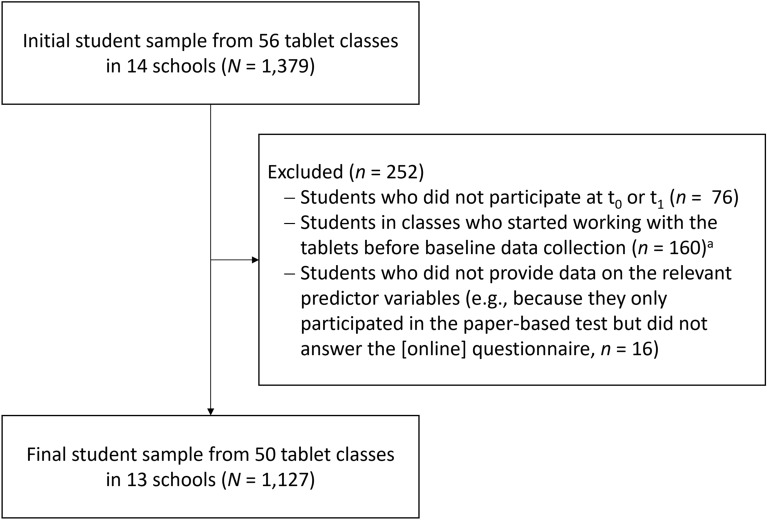
Student participant flowchart. (^a^Students of these classes were excluded because the change we were interested in had already taken place in these schools; the exclusion did not change the pattern of results.)

A total of 1,379 students from tablet classes participated in the data collections of the overall project. After applying our exclusion criteria (for student participant flow, see Fig. 2), the sample we analyzed comprised data from *N* = 1,127 students (548 female, 577 male, two students did not provide their gender) at the age of 11 to 17 years (*M* = 12.49 years, *SD* = 0.59, four students did not provide their age). The sample comprised 1,020 complete cases, 78 cases that participated only at t_0_, and 29 cases that participated only at t_1_.

#### Measures

All items of the students’ questionnaire scales relevant to this study are listed in Appendix B.

Regulatory orientation was assessed as promotion focus (Cronbach’s α = 0.77, e.g., “I want to achieve a great deal in my life.") and prevention focus (Cronbach’s α = 0.71, e.g., “In case of important decisions, security is important to me.”), each being measured with six items at baseline (t_0_). These items were adapted from the scale used in Study 1^34^, originally designed for adults. Items suitable for students were selected, and some items were adapted to better fit students’ lives in school (e.g., “Success sets me at ease.” from the original scale was changed to “If I receive a good grade, it reassures me.”). All items were measured on a 5-point scale from (1) *strongly disagree* to (5) *strongly agree*.

Perception of technology (10 items of which five items were reversely coded; Cronbach’s α = 0.76; e.g., “I like to use digital devices.”), technology-related self-efficacy (7 items of which one item was reversely coded; Cronbach’s α = 0.82; e.g., “If a problem arises with a digital device, I think I can solve it.”), and motivation to use technology (7 items; Cronbach’s α = 0.75; e.g., “I like to familiarize myself with new digital devices."; motivation at t_0_ was mistakenly assessed on a 5-point scale; thus, for the purpose of this study, the values of each item were rescaled to a 4-point scale.) were each measured on 4-point scales ranging from (1) *not at all* to (4) *very much* (adapted and extended from the scales interest in ICT, personally perceived ICT competence, and perceived autonomy in ICT use from ^66^). The three scales were measured at both measurement points, t_0_ and t_1_. For the sake of brevity, these variables hereafter are referred to as perception, self-efficacy, and motivation, respectively.

#### Procedure

Trained research assistants conducted data collection in the classrooms at each measurement point. Given that consent was provided by the students and their legal guardians, the students completed the questionnaires online on their tablets. The self-report questionnaires comprised questions on how students worked with the tablet computers (e.g., frequency of use per subject), how they perceived their learning environment in classes with and without tablet computers (e.g., perceived teaching quality), and students’ backgrounds (e.g., demographics). Furthermore, paper–pencil instruments tested students’ abilities (e.g., cognitive abilities). The scales relevant to the current research (i.e., regulatory orientation, perception of technology, technology-related self-efficacy, motivation to use technology) were all assessed in the online self-report questionnaire. Rather stable variables were only assessed at t_0_ (e.g., personality), whereas variables expected to vary over time were assessed at each measurement point (e.g., perception of digital media). At t_0,_ data collection took 180 min and was divided into two sessions of 90 min each. Each following measurement point lasted 90 min.

### Results

Again, the analyses were conducted with the statistical software R version 4.3.1^41^ and the lavaan package version 0.6–15^42^. Descriptive values (means and standard deviations) and correlations of all study variables are reported in Table 3.

**Table 3:** Descriptive statistics (sample size, mean, standard deviation) and zero-order correlations for all study variables in the student sample.

	*n*	*M*	*SD*	1	2	3	4	5	6	7
1. Promotion focus (t_0_)	980	4.34	0.55	–						
2. Prevention focus (t_0_)	980	3.96	0.61	0.39***	–					
3. Perception (t_0_)	1001	2.72	0.52	0.13***	− 0.17***	–				
4. Self-efficacy (t_0_)	1001	2.95	0.56	0.28***	− 0.01	0.55***	–			
5. Motivation (t_0_)	1001	2.94	0.54	0.25***	< 0.01	0.53***	0.72***	–		
6. Perception (t_1_)	1038	2.68	0.43	0.10**	− 0.02	0.51***	0.32***	0.31***	–	
7. Self-efficacy (t_1_)	1038	2.89	0.56	0.20***	− 0.01	0.43***	0.63***	0.49***	0.46***	–
8. Motivation (t_1_)	1038	2.86	0.56	0.16***	− 0.04	0.39***	0.50***	0.52***	0.35***	0.69***

*Note*. Promotion and prevention focus were assessed on a 5-point scale; perception, self-efficacy, and motivation were assessed on a 4-point scale. ***p < 0.001, **p < 0.01, *p < 0.05.

We investigated the hypotheses that students’ promotion focus at the time they got the tablet computers (t_0_) would be positively associated with their perception, self-efficacy, and motivation at the end of the school year (t_1_; hypothesis 1a, 2a, 3a) and that students’ prevention focus at the time they got the tablet computers (t_0_) would be negatively associated with their perception, self-efficacy, and motivation at the end of the school year (t_1_; hypothesis 1b, 2b, 3b). To this end, we fitted a regression model (Table 4) to examine the effects of promotion focus and prevention focus both at t_0_ on students’ perception, self-efficacy, and motivation each at t_1_ while controlling for the student’s perception, self-efficacy, and motivation at t_0_, respectively, as well as for age and gender (female = 0, male = 1). Interaction terms were not included in the model. The parameters were calculated using maximum likelihood estimation using the Yuan-Bentler correction and cluster robust standard errors with class as cluster variable^43^. Further, we estimated the variances and covariances of the exogenous variables in the model to account for these variables when estimating missing values in the full information maximum likelihood procedures for missing data.

**Table 4:** Fitted model of the student sample.

Predictor	Dependent variables								
	Perception (t_1_)			Self-efficacy (t_1_)			Motivation (t_1_)		
	*Est*	*SE*	*p*	*Est*	*SE*	*p*	*Est*	*SE*	*p*
Promotion focus (t_0_)	0.016	0.024	0.505	0.079	0.032	**0.014**	0.090	0.039	**0.022**
Prevention focus (t_0_)	0.032	0.021	0.133	− 0.020	0.032	0.520	− 0.051	0.033	0.116
Perception (t_0_)	0.399	0.052	** < 0.001**						
Self-efficacy (t_0_)				0.489	0.026	** < 0.001**			
Motivation (t_0_)							0.413	0.039	** < 0.001**
Gender	− 0.063	0.030	**0.038**	0.089	0.033	**0.006**	0.155	0.037	** < 0.001**
Age	0.003	0.022	0.909	< 0.001	0.025	0.990	− 0.006	0.027	0.838
*R*^*2*^	0.226			0.336			0.250		
*Inferential statistical safeguarding of the model quality*									
χ^2^	70.91, *p* < 0.001								
*df*	6								
SRMR	0.041								
CFI	0.980								
RMSEA	0.098								

*Note. N* = 1127. Significant *p*-values (*p* < 0.05) are shown in bold. Following Hu and Bentler^67^, we used the Chi-Square Test of Model Fit (χ^2^), the Standardized Root Mean Square Residual (SRMR), the Comparative Fit Index (CFI), and the Root Mean Square Error of Approximation (RMSEA) to evaluate the model fit. The predicted model showed an acceptable fit to the data.

#### RQ: Is students’ regulatory orientation related to their motivational outcomes in the context of introducing technology-enhanced teaching?

In line with expectations, the results (see Table 4) showed a positive association of students’ baseline promotion focus (t_0_) with their self-efficacy (hypothesis 2a) and motivation (hypothesis 3a) after working with tablet computers in school for some months (t_1_). Other than expected, however, this relation was not observed for students’ perception of technology (hypothesis 1a). Further, students’ prevention focus at baseline neither showed a relation to their perception (hypothesis 1b), self-efficacy (hypothesis 2b), or motivation (hypothesis 3b) after working with tablet computers in school for some months (t_1_).

Further, the model showed significant differences for gender. After having worked with tablet computers in school for some months, male compared with female students perceived technology less positively, but had higher self-efficacy beliefs, and were more motivated to use technology.

### Discussion

Study 2 shed light on how students dealt with changing teaching practices from switching to technology-enhanced teaching and learning. It supported our hypothesis that students’ promotion focus was associated with motivational consequences of introducing tablet computers into the classroom. In contrast and similar to the teachers’ results, students’ prevention focus did not play a role. Thus, the results can be considered as further evidence of the relevance of individuals’ promotion focus when introducing technology in classrooms and that prevention focus is of minor relevance.

It should be noted, however, that we examined motivational outcomes related to technology as a dependent measure. That students feel more competent in using technology (stronger self-efficacy beliefs) or are more motivated to use technology is just a first step for successful technology integration. As reported earlier, motivational variables are closely related to other outcome measures^58,60^. However, future research should investigate whether more favorable motivational conditions induced by promotion focus are then associated with high-quality technology use and higher learning achievement.

Inconsistent with the other findings of teachers’ or students’ promotion focus, there was no relation between students’ baseline promotion focus and later perception of technology. However, gender effects also appeared in the opposite direction for perception compared to the gender effects of the other criterion variables, self-efficacy and motivation. Thus, it remains to be investigated in further research whether student promotion focus is indeed unrelated to the perception of digital media or whether the inconsistent pattern of findings was caused, for example, by difficulties in measuring students’ perception.

## General discussion

The presented research aimed to investigate regulatory orientation as a possible determinant of successful change processes in schools. To this end, we examined teachers’ and students’ promotion and prevention focus as predictors of successfully dealing with the changes occurring from the introduction of technology into the classroom. In line with expectations, we showed that promotion focus related to teachers’ intention to use tablets in the classroom and to students’ technology-related self-efficacy and motivation to use digital media; contrary to expectations, prevention focus did not relate to any outcome measures for both teachers and students.

To begin with, our findings show that regulatory focus theory can be applied to the educational context. We made use of data from a research project which evaluated the introduction of tablet computers to the classroom and tied in with a school trial initiated by the Ministry of Cultus, Youth, and Sports in Baden-Württemberg, Germany. In this vein, we considered the school context as both a workplace for teachers and an educational environment for students. We showed coherent patterns of results across both samples that are consistent with findings from other fields^9,21^. This can be considered further evidence for the generalizability of regulatory focus theory to various contexts.

Moreover, our results show that an individual’s promotion focus and the accompanying openness to new situations seem relevant for switching to technology-enhanced teaching. Prevention focus and the accompanying preference for stability do not seem pivotal in dealing with the introduction of technology in the classroom. Thus, our results highlight promotion focus as a powerful predictor in change contexts, while the role of prevention focus requires further investigation. This implies that it might be helpful to consider the regulatory orientation of individuals involved when changes are pending at schools. Future research could investigate whether changes succeed better when the corresponding instruction is tailored to fit the regulatory orientation of the individuals involved. That is, individuals with a strong promotion focus are likely to prefer to be given the freedom to try out the new feature. In contrast, individuals with a strong prevention focus may prefer a gradual approach and might need more time to become familiar with the new feature.

In our research, we considered teachers and students separately. In a next step, it would be fruitful to look at the interplay between the regulatory orientation of teachers and students. Findings from the work context show differential effects depending on the regulatory fit between managers and employees on motivational variables and performance^63,65^. Similar mechanisms could be envisioned in the interpersonal interaction of teachers and students.

As a limitation, it should be mentioned that the sample was an opportunity sample from the pool of applicants for the school trial. The schools that received money to equip their classes with tablets were drawn randomly. However, it can be assumed that the teachers’ council agreed upon an application for the school trial and that mainly schools with a high affinity for technology applied. In addition, likely, teachers who were particularly motivated to teach with tablets were primarily deployed in the equipped classes. However, the effects of regulatory orientation should also be evident in self-selection samples because the mechanisms of regulatory orientation are supposed to work unconsciously^9^. Therefore, we assume that our findings are meaningful, nevertheless.

## Conclusion

In conclusion, findings from a German school trial suggest that regulatory focus theory can be applied to change processes in the educational context. In particular, promotion focus has been shown to be an influential determinant in dealing with change for both teachers and students. In the future, the fit of regulatory orientation to corresponding change measures or between involved stakeholders should be considered and merits further exploration.

## Supplementary Information

Supplementary Information 1.

Supplementary Information 2.

## Data Availability

The datasets generated and/or analyzed during the current study are available from the corresponding author upon reasonable request.
