# Supplementary material for: Promotion focus, but not prevention focus of teachers and students matters when shifting towards technology-based instruction in schools
Source: Sci Rep. 2024 Sep 25;14:22030. doi: 10.1038/s41598-024-73463-z (PMC11424645; doi:10.1038/s41598-024-73463-z)
Supplement: Supplementary file 1 — Supplementary Information 1. [file 41598_2024_73463_MOESM1_ESM.pdf]

Appendix A - Scales Used in the Teacher Questionnaire

Regulatory Orientation[34]

Promotion Focus

- I want to achieve a great deal.
- I am very productive.
- I am striving for success in live.
- I wholeheartedly go for my goals.
- My motto is "Nothing ventured, nothing gained".
- If I really want to achieve a goal, I will find a way.
- I am guided by my ideals.
- I like trying out new things.
- The big picture is more important to me than the details.
- At times I am fanatic about achieving my goals.
- I am ready to take risks.
- I am striving for progress.

Prevention Focus

- I am not a cautious person. (−)
- I am literally always following rules and regulations.
- In case of important decision security is a core criterion I care for.
- Every now and then I violate rules and regulations, to reach my goals. (−)
- Success sets me at ease.
- I take care to carry out my duties.
- If I do not reach my goal, I am becoming nervous.
- On the job and in my studies, thoroughness is important to me.

Intentions to Use Tablet Computers (self-developed)

- I intend to continue my professional development in the area of tablets in the future.
- I will use educational software in my subject lessons in the future.
- I will carry out an internet project in my subject lessons in the future.
- I will look for meaningful ways to use tablet computers in my classroom in the future.
- In the future, I will look for opportunities to increase the proportion of tablet computer use in my lessons.

Stress Appraisal Measure (adapted from [40])

<u>Challenge</u>

- I can handle this situation.
- I tackle the problems head on.
- I am excited about the outcome.
- The high task demands spur me on.
- I can use the high demands positively for my purposes.
- I always feel that I can handle this situation.
- I feel like I have become/am becoming stronger because of this situation.

<u>Threat</u>

- The high task demands put a lot of strain on me.
- I feel helpless.
- This situation stresses me out tremendously.
- I feel like I'm not in control of this situation.
- For me this situation is threatening.
- I am afraid of not being able to cope with this situation.
- This situation worries me very much.
