# Supplementary material for: Promotion focus, but not prevention focus of teachers and students matters when shifting towards technology-based instruction in schools
Source: Sci Rep. 2024 Sep 25;14:22030. doi: 10.1038/s41598-024-73463-z (PMC11424645; doi:10.1038/s41598-024-73463-z)
Supplement: Supplementary file 2 — Supplementary Information 2. [file 41598_2024_73463_MOESM2_ESM.pdf]

Appendix B—Scales Used in the Student Questionnaire

Regulatory Orientation (adapted from [34])

<u>Promotion Focus</u>

- I want to achieve a great deal in my life.
- If I really want to achieve a goal, I will find a way.
- I would like to be very successful in my life.
- I like trying out new things.
- When I do something, it is important for me to progress.
- I wholeheartedly go for my goals.

<u>Prevention Focus</u>

- If I receive a good grade, it reassures me.
- I am literally always following rules (e.g., classroom rules).
- I am a cautious person.
- In case of important decisions, security is important to me.
- In school, thoroughness is important to me.
- I take care to carry out my duties.

Perception of Technology (adapted from [66])

- I do not like to play computer or video games. (−)
- I prefer to do my homework without rather than with digital devices. (−)
- It is very useful to have social networks on the Internet.
- I don't understand how people could live before digital devices were invented.
- Hours of surfing the Internet is a waste of time. (−)
- When new digital devices come on the market, it's not important to me. (−)
- I don't want to spend money on expensive digital devices. (−)
- I get very excited when I discover new digital devices or applications.
- I feel really bad when I can't connect to the Internet.
- I like to use digital devices.

Technology-Related Self-Efficacy (adapted from [66])

- I am good at using digital devices.
- I also feel good about using digital devices that I am less familiar with.

- If friends or relatives want to buy new digital devices or applications, I can give them some advice.
- I feel good about using my digital devices at home.
- If a problem arises with a digital device, I think I can solve it.
- If my friends or relatives have a problem with a digital device, I can help them.
- When a digital device causes problems, I feel rather helpless. (−)

Motivation to Use Technology (adapted from [66])

- I like to familiarize myself with new digital devices.
- When I need new software (programs), I install them myself.
- To be self-sufficient, I read information about digital devices.
- I use digital devices the way I want to use them.
- In my free time I follow topics related to digital devices.
- When I have a problem with a digital device, I start to solve it myself.
- When I need a new application, I select it myself.
